# Supplementary material for: Expectation violations signal goals in novel human communication
Source: Nat Commun. 2025 Feb 26;16:1989. doi: 10.1038/s41467-025-57025-z (PMC11865554; doi:10.1038/s41467-025-57025-z)
Supplement: Supplementary file 2 — Reporting Summary [file 41467_2025_57025_MOESM2_ESM.pdf]

nature portfolio

Corresponding author(s): Tatia Buidze

Last updated by author(s): Aug 9, 2024

Reporting Summary

Nature Portfolio wishes to improve the reproducibility of the work that we publish. This form provides structure for consistency and transparency in reporting. For further information on Nature Portfolio policies, see our Editorial Policies and the Editorial Policy Checklist.

Statistics

For all statistical analyses, confirm that the following items are present in the figure legend, table legend, main text, or Methods section.

n/a	Confirmed	
☐	☒	The exact sample size (*n*) for each experimental group/condition, given as a discrete number and unit of measurement
☐	☒	A statement on whether measurements were taken from distinct samples or whether the same sample was measured repeatedly
☐	☒	The statistical test(s) used AND whether they are one- or two-sided<br>*Only common tests should be described solely by name; describe more complex techniques in the Methods section.*
☐	☒	A description of all covariates tested
☐	☒	A description of any assumptions or corrections, such as tests of normality and adjustment for multiple comparisons
☐	☒	A full description of the statistical parameters including central tendency (e.g. means) or other basic estimates (e.g. regression coefficient) AND variation (e.g. standard deviation) or associated estimates of uncertainty (e.g. confidence intervals)
☐	☒	For null hypothesis testing, the test statistic (e.g. *F*, *t*, *r*) with confidence intervals, effect sizes, degrees of freedom and *P* value noted<br>*Give P values as exact values whenever suitable.*
☒	☐	For Bayesian analysis, information on the choice of priors and Markov chain Monte Carlo settings
☐	☒	For hierarchical and complex designs, identification of the appropriate level for tests and full reporting of outcomes
☒	☐	Estimates of effect sizes (e.g. Cohen's *d*, Pearson's *r*), indicating how they were calculated

Our web collection on statistics for biologists contains articles on many of the points above.

Software and code

Policy information about availability of computer code

Data collection	Behavioral data were acquired using the MATLAB software integrated with Psychtoolbox 3. Eye-tracking measurements were obtained via the iView Red-m system from Sensorimotor Instruments (SMI), Teltow, Germany. Electroencephalography (EEG) recordings were conducted using the 128-channel ActiveTwo system provided by BioSemi, based in Amsterdam, Netherlands. The experimental tasks were operated and computational model simulations were conducted using custom-developed code in Matlab. All related code is publicly accessible at the authors' GitHub page at https://github.com/TatiaBu/CommunicationSurprise_DataCode.git.
Data analysis	Eye-tracking data analysis: Eye tracking data was preprocessed by the guidelines and corresponding code (https://github.com/ElioS-S/pupil-size) suggested by Kret et al. The data analysis of eye-tracking data was conducted using lme4 package in R version 3.6.1.<br>EEG data analysis: The preprocessing steps were carried out using the EEGLAB software version 2022.1. The EEG data analysis was conducted using a cluster-based permutation test, as implemented in the fieldtrip software package.<br>Analyzing data generated from computational models: The data were analyzed using custom-made codes in Matlab. We used the Computational and Behavioral Modeling (CBM) toolbox (version 3, MATLAB R2021), which uses a variational Bayes approach to estimate the parameters of computational models<br>Statistical analysis: to test hypothesis we used the Matlab package for Bayesian hypothesis testing provided by Hoijtink et at.<br>All related code is publicly accessible at the authors' GitHub page at https://github.com/TatiaBu/CommunicationSurprise_DataCode.git.

For manuscripts utilizing custom algorithms or software that are central to the research but not yet described in published literature, software must be made available to editors and reviewers. We strongly encourage code deposition in a community repository (e.g. GitHub). See the Nature Portfolio guidelines for submitting code & software for further information.

Data

Policy information about availability of data

All manuscripts must include a data availability statement. This statement should provide the following information, where applicable:

- Accession codes, unique identifiers, or web links for publicly available datasets
- A description of any restrictions on data availability
- For clinical datasets or third party data, please ensure that the statement adheres to our policy

All behavioral and physiological data necessary to run the analysis are publicly available at https://doi.org/10.5281/zenodo.14333555. The EEG data generated in this study are available in the [database name] under accession code https://gin.g-node.org/glaescher/tcg.

Research involving human participants, their data, or biological material

Policy information about studies with human participants or human data. See also policy information about sex, gender (identity/presentation), and sexual orientation and race, ethnicity and racism.

Reporting on sex and gender	Dataset 1 comprised 41 female and 17 male participants, while Dataset 2 consisted of 35 female and 27 male participants. Selection of participants for both datasets was randomized from the general population, and consequently, no analysis based on sex was conducted.
Reporting on race, ethnicity, or other socially relevant groupings	NA
Population characteristics	See "Research sample" bellow
Recruitment	Study participants were recruited through university job boards. Exclusion criteria were a prior history of neurological or psychiatric diseases. All the participants were naive to the task and have been instructed with the standardized written instructions. All participants provided written informed consent prior to the study and were financially reimbursed for their participation. Each participant received a base payment of 12 euros for every hour of participation in the experiment. In addition to the base payment, participants were eligible for a bonus (Mean bonus: 4 euros) based on their performance.
Ethics oversight	The ethics committee of the General Medical Council of the City of Hamburg granted approval for this study (PV7114)

Note that full information on the approval of the study protocol must also be provided in the manuscript.

Field-specific reporting

Please select the one below that is the best fit for your research. If you are not sure, read the appropriate sections before making your selection.

☒ Life sciences ☐ Behavioural & social sciences ☐ Ecological, evolutionary & environmental sciences

For a reference copy of the document with all sections, see nature.com/documents/nr-reporting-summary-flat.pdf

Life sciences study design

All studies must disclose on these points even when the disclosure is negative.

Sample size	Dataset 1 involved 58 individuals (Mean age: M=25, SD=4.4), consisting of 29 pairs of Senders and Receivers, while dataset 2 included 72 participants (Mean age: M=26, SD=4.1), comprising 31 pairs of Senders and Receivers. Study participants were recruited through university job boards. Exclusion criteria were a prior history of neurological or psychiatric diseases. Dataset 1 comprised 41 female and 17 male participants, while Dataset 2 consisted of 35 female and 27 male participants. Selection of participants for both datasets was randomized from the general population, and consequently, no analysis based on sex was conducted. Our sample size was chosen to mirror successful practices in similar studies, adjusted for our specific research context and population, ensuring an optimal balance between statistical power and practical feasibility
Data exclusions	EEG data Exclusion: For the EEG data analysis we analyzed the Dataset 2 comprising 31 pairs of Receivers. EEG data were visually inspected to identify bad channels to be interpolated (average number of rejected channels: 6; Total number of channels: 128) and bad epochs to be rejected (average number of rejected epochs: 40; Average total number of epochs: 550). Blinks were removed by using Independent Component Analysis (average number of removed components: 5). We completely excluded 3 participants from the final data analysis because of poor data quality. PDR data exclusion: For the PDR data analysis we analyzed Dataset 1 comprising 29 datasets of the Receiver. After preprocessing we excluded 6 participants from the analysis because of the poor data quality (for these participants, more than 60 percent of the data was removed during the preprocessing). Behavioral Data Exclusions: For the behavioral data analysis there was no need to exclude any participants.

Replication	All attempts to reproduce the data were successful and there is no part of the results that can not be reproduced.
Randomization	The participants were allocated into the experimental groups randomly.
Blinding	In our study involving a 'sender' and 'receiver' players, we didn't blind investigators to participant. This was because the study design required observing real-time, interactive learning. Roles were randomly assigned to maintain fairness and reduce bias, making blinding unnecessary and impractical for our aims.

Reporting for specific materials, systems and methods

We require information from authors about some types of materials, experimental systems and methods used in many studies. Here, indicate whether each material, system or method listed is relevant to your study. If you are not sure if a list item applies to your research, read the appropriate section before selecting a response.

Materials & experimental systems

n/a	Involved in the study
☒	☐ Antibodies
☒	☐ Eukaryotic cell lines
☒	☐ Palaeontology and archaeology
☒	☐ Animals and other organisms
☒	☐ Clinical data
☒	☐ Dual use research of concern
☒	☐ Plants

Methods

n/a	Involved in the study
☒	☐ ChIP-seq
☒	☐ Flow cytometry
☒	☐ MRI-based neuroimaging

Plants

Seed stocks	NA
Novel plant genotypes	NA
Authentication	NA
